# *Entamoeba gingivalis*: epidemiology, genetic diversity and association with oral microbiota signatures in North Eastern Tanzania

**DOI:** 10.1080/20002297.2021.1924598

**Published:** 2021-05-19

**Authors:** Christen Rune Stensvold, Michelle Nielsen, Vito Baraka, Rolf Lood, Kurt Fuursted, Henrik Vedel Nielsen

**Affiliations:** a Department of Bacteria, Parasites and Fungi, Statens Serum Institut, Copenhagen S, Denmark; b Department of Biomedical Sciences, National Institute for Medical Research, Tanga Centre, United Republic of Tanzania; c Department of Clinical Sciences, Division of Infection Medicine, Lund University, Lund, Sweden

**Keywords:** Africa, sub-saharan africa, amoeba, ngs, oral microbiota, parasite, parasitology, periodontitis

## Abstract

**Background:**

*Entamoeba gingivalis* has been associated with periodontal diseases. Baseline data from the background population, which could help delimit the role of the parasite in health and disease, remain limited.

**Objective:**

To describe epidemiological features, genetic diversity, and associations with oral microbiome signatures of *E. gingivalis* colonisation in Tanzanians with non-oral/non-dental diseases.

**Methods:**

DNAs from 92 oral washings from 52 participants were subject to metabarcoding of ribosomal genes. DNA sequences were identified to genus level and submitted to oral microbiota diversity analyses.

**Results:**

Sixteen (31%) of the 52 study participants were *E. gingivalis-*positive, with no difference in positivity rate according to gender or age. Only one subtype (ST1) was found. Individuals testing positive for *E. gingivalis* had higher oral microbiota alpha diversity than those testing negative (*P* = 0.03). Eight of the top-ten most common bacterial genera were shared between the two groups (*Alloprevotella, Fusobacterium, Gemella, Haemophilus, Neisseria, Porphyromonas, Prevotella, Streptococcus*, and *Veillonella)*. Meanwhile, *E. gingivalis* carriers and non-carriers were more likely to have *Aggregatibacter* and *Rothia*, respectively, among the top-ten most common genera.

**Conclusion:**

About one third of the cohort carried *E. gingivalis* ST1, and carriers had higher oral microbiome diversity and were more predominantly colonized by *Aggregatibacter*.

## Introduction

The genus *Entamoeba* comprises several species known to colonise the human gastrointestinal tract [1,2]. Most of these produce cysts; however, for *Entamoeba gingivalis*, a cyst stage remains to be confirmed. This species is found mainly in the oral cavity, but has also been found in samples from genital tracts of intrauterine contraceptive device users [3]. Transmission occurs via contaminated food or mouth utensils, mouth droplets and kissing [4,5]. *E. gingivalis* may be a direct or indirect cause of periodontal disease [4,6–8], a condition affecting about 538 million people in 2015 [9], and which is a frequent cause of edentulism.

A handful of PCR-based surveys of *E. gingivalis* have been published [4,5,7,10–12], and very limited data exist on the prevalence of *E. gingivalis* in the background population; such data could serve to further elucidate the role of the parasite in oral health and disease.

At least two subtypes of *E. gingivalis* have been described, ST1 and ST2, which may co-exist, and, although data are scarce, they appear not to exhibit any particular demographical, geographical or clinical clustering [4,13,14]. These two subtypes differ substantially across the nuclear small subunit *rRNA* gene and might be considered different species, given the recent insight into the phylogeny of *Entamoeba* [1,2].

To date, only one study [15] sought to investigate whether the oral bacterial microbiota is correlated to *E. gingivalis* colonisation status; however, this study only included study participants referred for endodontic treatment.

In order to contribute baseline data on *E. gingivalis* carrier status in the background population, the aims of the present study were therefore to i) reveal epidemiological features of *E. gingivalis* colonisation in a cohort of Tanzanian individuals consulting their general practitioners for non-oral or non-dental diseases, ii) explore the genetic diversity within *E. gingivalis* in the sample set, and iii) identify whether *E. gingivalis* colonisation is associated with oral microbiota diversity patterns in a cohort of Tanzanian individuals.

## Materials and methods

### Sample collection and DNA extraction

A cross-sectional study was carried out in June 2019 at the Tanga Regional Referral Hospital, Tanzania. The study was approved by the Medical Research Coordinating Committee of the National Institute for Medical Research (NIMR MRCC; reference number, NIMR/HQ/R8.a/Vol.IX/3079). All individuals signed a written informed consent before enrolment in the study. The study enrolled 26 patients, who had been prescribed oral antibiotics (3–10 days) for treatment of non-oral infections, and 26 patients at the hospital who had not received antibiotics (*e.g*. non-infectious reasons for hospitalization for 3–10 days). The latter group had no history of antibiotic treatment in the past three months. Saliva samples (5 mL) were collected on day 0 (hospitalisation day) and day 3, and DNA was isolated using Norgen’s Saliva DNA preservation and isolation kit (Norgen Biotek Corp., Thorold, ON, Canada). For all samples, information on age, gender, and treatment was available. Samples collected from the control individuals are referred to as ‘no-treatment samples’, while those from patients offered treatment are referred to as ‘pre-treatment samples’ and ‘post-treatment samples’ for day 0 and day 3, respectively.

### Oral microbiota profiling

Genomic DNAs extracted from the mouthwash samples were subject to microbiota profiling (metabarcoding), using the recently described 16S + 18S assay in place at Statens Serum Institut [16–18]. Briefly, PCR used one primer pair targeting the 16S (341F3/806R5 [5ʹ-ACT CCT AYG GGR BGC ASC AG-3ʹ/5ʹ-AGC GTG GAC TAC NNG GGT ATC TAA T-3ʹ]) and three primer pairs targeting the 18S (G3F1/G3R1 [5ʹ-GCC AGC AGC CGC GGT AAT TC-3ʹ/5ʹ-ACA TTC TTG GCA AAT GCT TTC GCA G-3ʹ], G4F3/G4R3 [5ʹ-CAG CCG CGG TAA TTC CAG CTC-3ʹ/5ʹ-GGT GGT GCC CTT CCG TCA AT-3ʹ], and G6F1/G6R1 [3ʹ-TGG AGG GCA AGT CTG GTG CC-3ʹ/5ʹ-ACG GTA TCT GAT CGT CTT CGA TCC C-3ʹ]). DNA was quantified using the Quant-IT high-sensitivity double-stranded DNA (dsDNA) assay kit (Thermo Fisher Scientific), and the PCR products were pooled in equimolar amounts from the individual samples into the pooled amplicon library (PAL). Undesirable DNA amplicons were removed from the PAL using Agencourt AMPure XP Bead (Beckman Coulter)-based purification as previously described [16]. The resulting AMPure bead-purified PAL was diluted to its final concentration of 11.5 pM DNA with a 0.001 N NaOH concentration and used for sequencing on the Illumina MiSeq desktop sequencer (Illumina Inc., San Diego, CA, USA). The library was sequenced with the 500-cycle MiSeq reagent kit, V2, in a 2 x 250-nucleotide setup (Illumina Inc., San Diego, CA, USA).

Annotation of sequence reads to taxonomic level was performed using BION (http://box.com/bion). The pipeline accepts raw sequences and includes steps for demultiplexing, primer extraction, sampling, sequence- and quality-based trimming and filtering, dereplication, clustering, chimera checking, identification of similarities to reference data, and taxonomic mapping and formatting. Non-overlapping paired reads were allowed for analysis. The assay enables the detection and differentiation of both of the subtypes (ST1 and ST2) of *E. gingivalis* in both oral and bronchial lavage samples (data not shown); however, the analytical and diagnostic sensitivity of the assay for the detection of *E. gingivalis* remains unknown.

### Data analyses

Data were summarized as means and medians with standard deviations and interquartile ranges, respectively. Fisher’s Exact and unpaired *t* tests were carried out using the free online version of Prism by GraphPad (https://www.graphpad.com/). *Entamoeba*-specific DNA sequences were clustered using Clustal Omega (https://www.ebi.ac.uk/Tools/msa/clustalo/), and consensus sequences were generated after manual inspection. The genotype of *E. gingivalis* was determined by simple BLAST query of *E. gingivalis-*specific consensus DNA sequences against the NCBI database. A representative sequence was submitted to GenBank with the accession number MW676260.

The BION server not only allows the extraction of FASTA files that can be used to query DNA sequences of interest against the NCBI Database, but also extraction of read counts per taxon per sample, which we used to carry out analyses in R (v 4.0.3) and R Studio (R Foundation for Statistical Computing, Vienna, Austria) R Core Team. The R package PhyloSeq was used to describe the sequencing data. A rarefaction threshold was set at 272 for non-fungal eukaryotes, 55 for fungi, and 21,936 for bacteria, respectively. Alpha diversity was illustrated with a ggplot, and individual differences were assessed with Shannon’s Diversity Index and compared between groups using the Mann–Whitney rank-sum test. Overall diversity and beta diversity were compared using principal coordinate analysis (PCoA) based on Bray–Curtis dissimilarity between samples. The effect of *E. gingivalis* on the abundance of bacterial taxa was analysed at genus level using the R package DESeq2 on raw sequence counts. The cut-off for Cook’s distance, a diagnostic test for outlier detection, was set to 0.99 (default) in the replace Outliers () function for all DESeq2 models. Heatmaps were used to detect the top-10 most common bacterial genera. A probability (*P*) value < 0.05 was considered to indicate statistical significance. Linear discriminant analysis Effect Size (LEfSe) [19] was used to identify and visualize the taxonomic differences between the different groups in the study.

## Results

### Samples

A total of 92 samples from 52 individuals were available for the study, and most individuals had submitted two samples; one at day 0 and one at day 3. A total of 16 individuals out of 26 who had been prescribed antibiotics had in fact received antibiotic treatment for non-oral infections and submitted a pre-treatment and a post-treatment sample.

### E. gingivalis *positivity rate and genetic diversity within* E. gingivalis

A total of 26 samples from 16 individuals were positive for *E. gingivalis* (Table 1), and only one of the two subtypes, ST1, was detected (GenBank accession no., MW676260). Antibiotic treatment did not appear to influence the positivity rate, since the proportion of samples reflecting post-antibiotic treatment did not differ from that observed in the pre-treatment and non-treatment samples (25% vs. 29%; *P* = 0.77, Fisher’s Exact Test). The age range of the study participants was 17–83 years, with a mean (SD) age of *E. gingivalis*-positive and -negative individuals of 33.19 (14.54) and 30.64 (14.31), respectively; no difference in age was observed between E. *gingivalis*-positive and -negative individuals (*P* = 0.56, unpaired *t* test). None of the two registered genders were more prone to infection (*P* = 0.76, Fisher’s Exact Test; Table 1).

**Table 1.** *E. gingivalis* colonisation according to treatment and time

	Antibiotic treatment received		No antibiotic treatment received		
	Time point 1: Pre-treatment	Time point 2: Post-treatment	Time point 1	Time point 2	Total
*Entamoeba gingivalis*-positive	8 (32%)	4 (25%)	7 (27%)	7 (28%)	26 (28%)
*Entamoeba gingivalis*-negative	17 (68%)	12 (75%)	19 (63%)	18 (62%)	66 (32%)
Total	25	16	26	25	92

*All participating individuals were invited to submit a sample on two different time points; however, some participants submitted only one sample.

A single example of polymorphism was observed across the sequences (see https://github.com/Entamoeba/Tanz_mouthwash), namely a T/G polymorphism, which was already pointed out in ST1 sequences by Garcia and colleagues [13].

### Bacterial diversity according to sample type (no treatment, pre-, or post-treatment)

Crude bacterial richness and alpha diversity of the pre-treatment, post-treatment, and non-treatment samples did not differ (*P* > 0.05 for all; Supplementary Figures 1 and 2). Meanwhile, pre- and post-treatment samples differed significantly in terms of beta diversity (*P* = 0.02; Supplementary Figure 3).

### *Bacterial diversity according to* E. gingivalis *carrier status*

**Figure 1.**
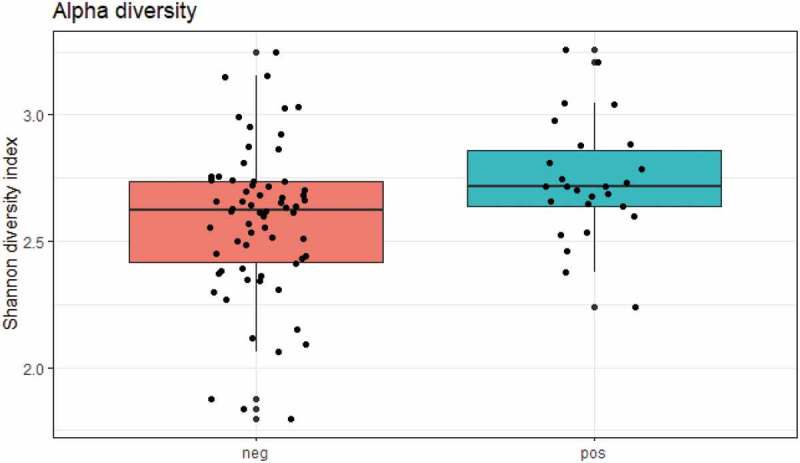
Overall alpha diversity as measured by Shannon’s Diversity Index in *E. gingivalis*-negative (coral red) and *E. gingivalis*-positive (turquoise) individuals (*P* = 0.03), including post-treatment samples

**Figure 2.**
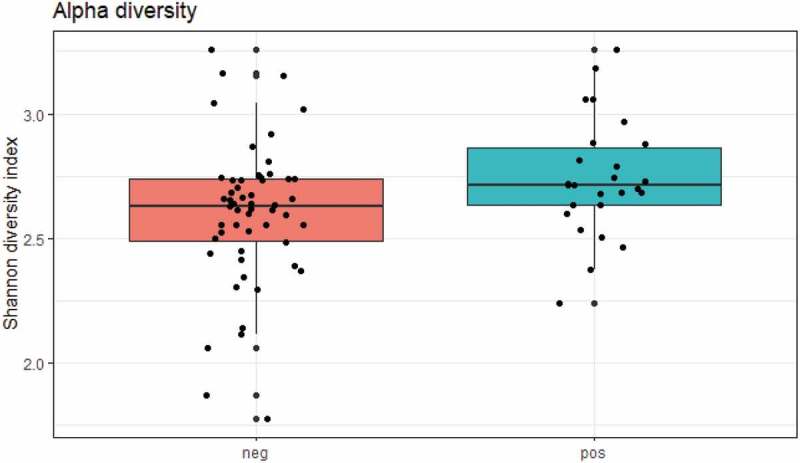
Overall alpha diversity as measured by Shannon’s Diversity Index in *E. gingivalis*-negative (coral red) and *E. gingivalis*-positive (turquoise) individuals (*P* = 0.03), excluding post-treatment samples (see text for details)

**Figure 3.**
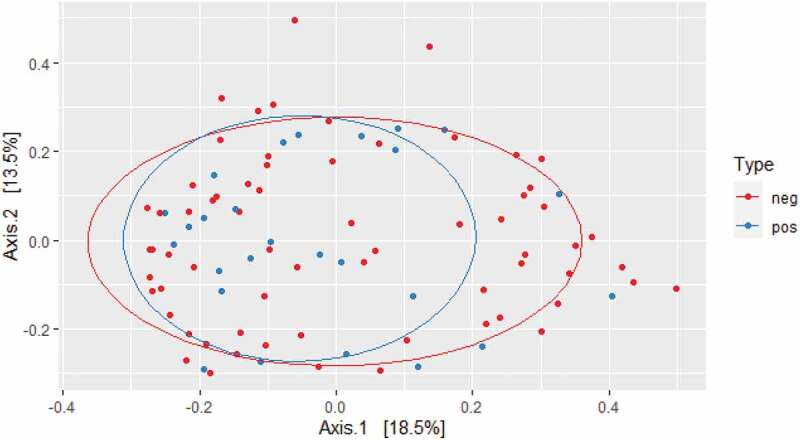
Beta diversity of *E. gingivalis*-negative (red dots) and *E. gingivalis*-positive (blue dots) individuals (*P* = 0.88) as measured by Bray–Curtis dissimilarity analysis

While crude bacterial richness based on the raw sequence count was similar in carriers and non-carriers, bacterial alpha diversity was significantly higher in *E. gingivalis*-positive individuals compared with *E. gingivalis*-negative individuals, regardless of treatment (*P* = 0.03) (Figure 1). Even when the 16 post-antibiotic treatment samples were excluded, alpha diversity was still higher in the *E. gingivalis*-positive samples (*P* = 0.03; Figure 2). No difference was observed in beta diversity between these two groups (*P* = 0.88), (Figure 3); however, the oral microbiomes of *E. gingivalis*-positive individuals were more homogenous than those of non-colonized individuals.

Looking only at individuals who were not offered treatment, crude bacterial richness did not differ between *E. gingivalis* carriers and non-carriers (Supplementary Figure 4), while alpha diversity was significantly higher among the carriers (Supplementary Figure 5), confirming the overall trend. Meanwhile, beta diversity between the carriers and non-carriers did not differ (Supplementary Figure 6), again confirming the overall trend.

The top-ten most common genera detected in the two groups are listed in Table 2. Eight of these 10 genera were shared between carriers and non-carriers. Meanwhile, non-carriers had *Rothia* among the top-ten most common genera, whereas carriers had *Aggregatibacter* among the top-ten most common genera. Removing the sixteen samples that reflected post-treatment samples from the data set did not affect the results of this analysis (data not shown).

**Table 2.** The top-ten most common genera identified in *E. gingivalis*-negative and *E. gingivalis*-positive individuals, respectively, as detected by heat map analysis. Genera that differ are indicated in boldface type

*Entamoeba gingivalis*-negative	*Entamoeba gingivalis*-positive
-	***Aggregatibacter***
*Alloprevotella*	*Alloprevotella*
*Fusobacterium*	*Fusobacterium*
*Gemella*	*Gemella*
*Haemophilus*	*Haemophilus*
*Neisseria*	*Neisseria*
*Porphyromonas*	*Porphyromonas*
*Prevotella*	*Prevotella*
***Rothia***	-
*Streptococcus*	*Streptococcus*
*Veillonella*	*Veillonella*

**Figure 4.**
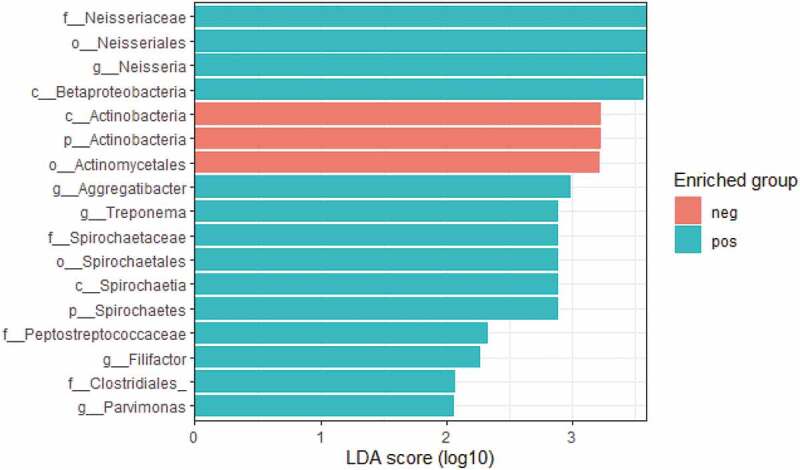
LEfSe plot displaying the bacterial taxa enriched in the *E. gingivalis*-positive (turquoise) and *E. gingivalis*-negative (coral red) groups, respectively

LEfSe revealed that the genera specifically enriched in *E. gingivalis*-positive individuals were *Neisseria, Aggregatibacter, Treponema, Parvimonas*, and *Filifactor*, while bacteria of the order *Actinomycetales* were enriched in the *E. gingivalis*-negative group (Figure 4).

## Discussion

In the present study, we identified *E. gingivalis* in 31% of Tanzanian study participants by metabarcoding of ribosomal genes in genomic DNA extracted from mouth wash samples and thereby provided the first baseline data on *E. gingivalis* colonisation in Sub-Saharan Africa.

To our knowledge, this is only the third study to provide data on *E. gingivalis* in the African subcontinent. Testing gum-line scrapings, Arene found an overall prevalence of 6.9% among inhabitants of the Nile Delta [20]. The other study with African participants was also from Egypt, but that study mainly focused on *E. gingivalis* identified in the genital tract of intrauterine contraceptive device users [3].

According to Garcia et al., *E. gingivalis* positivity rates among individuals with periodontal disease range between 30% and 81% [14], and even positivity rates as low as 12% have been identified [21]. These vast differences in positivity rates might reflect differences in diagnostic sensitivity among the methods used. In this study, we used DNA-based technology, which offers greater sensitivity than non-DNA-based methods, such as microscopy or culture, also in the case of *E. gingivalis* [5,11]. More data are needed, but the positivity rate observed in the present study could be a preliminary indication that *E. gingivalis* might be less common in the background population than in patients with periodontal disease. It should be noted that the diagnostic sensitivity of the assay used here has not undergone validation for the detection of *E. gingivalis* in mouth wash samples, and the sampling (mouth wash) might be less appropriate than gum scraping; hence, the 31% positivity rate identified in the present study might reflect a rather conservative estimate.

We did not identify any links between basic demographic features such as reported for age and gender. Arene and colleagues observed a decrease in prevalence by age; thus, 22.9% of the 5–10 year-olds were colonized as opposed to only 5.7% of those aged 29 or more [20]. In the present study, the youngest participant was 17 years, so this cohort could not be used to confirm or challenge the observation by Arene et al. A study from Iran found a significantly higher prevalence among men than among women [5]; however, other studies have failed to identify gender- or age-based differences [22].

This is the first study reporting *E. gingivalis* subtype data from the African continent. Of the two subtypes of *E. gingivalis* reported to date, only ST1 was identified in the present study. No inter-sequence variation was observed among the consensus sequences generated (data not shown). Dubar and colleagues identified *E. gingivalis* only in 1/30 (3.3%) of control individuals, and the subtype was ST1 [7]; meanwhile, both ST1 and ST2 were readily detected in patients with periodontitis. Garcia et al. [14] studied the distribution of *E. gingivalis* subtypes in individuals in Mexico with and without periodontal disease and in individuals undergoing orthodontic treatment, and identified both ST1 and ST2 in all three categories, albeit of various frequency. In those with no periodontal disease, ST1 was 1.64 x more prevalent than ST2 and was found in 48.6% of the individuals. In that study, nested PCR was used for detection, and this diagnostic approach might provide an even higher sensitivity than that of the single-round PCR used in the present study.

*E. gingivalis* has been suggested to be a direct or indirect contributor to dental-oral disease [4,6–8]; as an indirect contributor, it could increase the risk of secondary bacterial infection or exert predation on the oral microbiome, which could potentially select for a less favourable oral microbiome. Indeed, the higher bacterial diversity observed among those who were colonised by *E. gingivalis* could suggest predation by *Entamoeba* on oral bacteria, as amoebic predation on bacteria has been linked to higher diversity [23]. Interestingly, higher microbiome diversity was also seen among those with gastrointestinal colonisation by other species of *Entamoeba* [24]. While high microbiota diversity of the gut appears to be conducive to gut health [25], the reverse appears to be true for other organs, such as the vagina, and high microbiota diversity in the vagina may reflect vaginosis/dysbiosis [25,26] and make the vagina more susceptible to *e.g*. human papillomavirus infection [27]. Little is known, however, about the clinical significance of a higher-diversity oral microbiome.

*Aggregatibacter* and *Neisseria* spp. were enriched in samples testing positive for *E. gingivalis*, which is in line with the study by Koller et al., in which both genera were among those enriched in patients with *E. gingivalis*. Species of *Aggregatibacter* may be involved in periodontal disease [28–30]. Other genera, such as *Streptococcus, Veilonella*, and *Haemophilus* were also enriched in *E. gingivalis* carriers in the study by Koller et al. [15]; however, all three of these were among the top-ten most common genera in both carriers and non-carriers in the present study and did not differ specifically in abundance among the carriers and non-carriers.

Both *Parvimonas* and *Filifactor*, which were both enriched in *E. gingivalis*-positive samples in the present study, have been associated with periodontal disease (*Parvimonas micra* and *Filifactor alocis* [31,32]) and endodontic lesions (*F. alocis* [31]), indicating that positivity for *E. gingivalis* may be associated with enrichment for some bacteria that can cause such conditions. This hypothesis should be confirmed in future studies of other cohorts.

Limitations of the study include the fact that the sample size was small, and no children were included. However, one of the strengths is the fact that this cohort reflected individuals who had been submitting mouthwash samples due to reasons other than oral/dental diseases, and the data are therefore valuable as reference data.

### Conclusion

This study provided the first data on *E. gingivalis* carrier status in Sub-Saharan Africa. About one third of the cohort tested positive for *E. gingivalis* ST1, and those who tested positive had higher oral microbiome diversity and were more predominantly colonized by *Aggregatibacter* spp. Beta diversity among carriers and non-carriers did not differ.

## Supplementary Material

Supplemental Material

## Data Availability

The demographic data that support the findings of this study are available on request from the corresponding author, CRS. The data are not publicly available due to restrictions in place serving not to compromise the privacy of the research participants.

## Data deposition

One DNA sequence was uploaded to the NCBI database (MW676260). A repository of the *E. gingivalis* partial SSU rDNA sequences obtained in the study is available at https://github.com/Entamoeba/Tanz_mouthwash
