# Supplementary material for: Entamoeba gingivalis: epidemiology, genetic diversity and association with oral microbiota signatures in North Eastern Tanzania
Source: J Oral Microbiol. 2021 May 19;13(1):1924598. doi: 10.1080/20002297.2021.1924598 (PMC8143617; doi:10.1080/20002297.2021.1924598)
Supplement: Supplemental Material [file ZJOM_A_1924598_SM5356.docx]

Supplementary file.


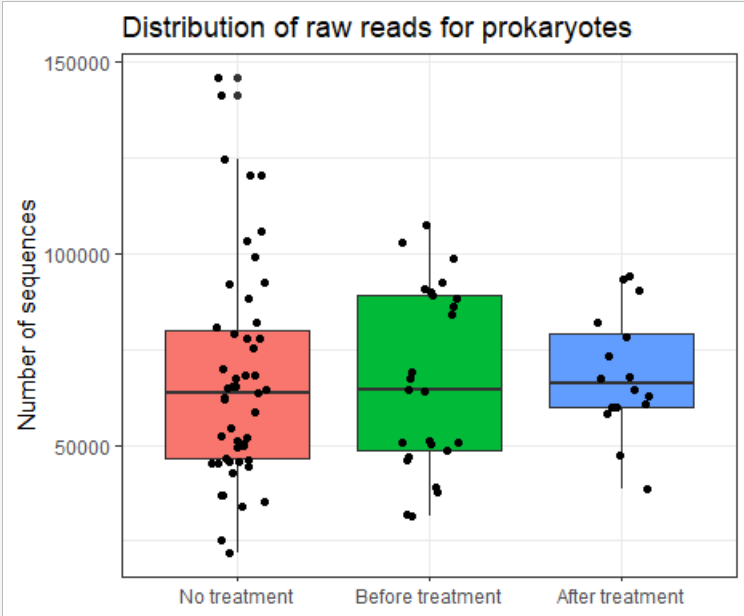


| *P* values | No treatment | Before treatment | After treatment |
| --- | --- | --- | --- |
| No treatment | NA | 0.7907482 | 0.4314196 |
| Before treatment | 0.7907482 | NA | 0.7412027 |
| After treatment | 0.4314196 | 0.7412027 | NA |

**Supplementary Figure 1.** Comparison of sample type (no treatment, vs. pre-treatment vs. post-treatment samples) with regard to the distribution of raw reads. NA = not applicable.


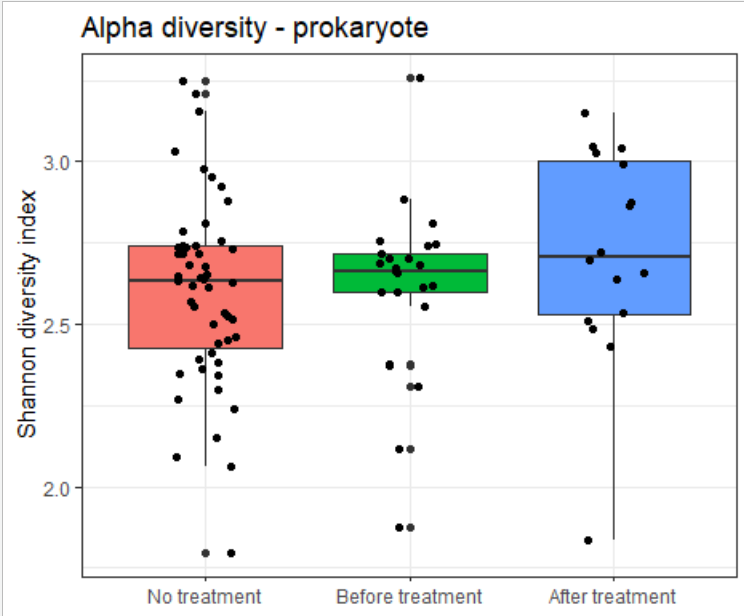


| *P* values | No treatment | Before treatment | After treatment |
| --- | --- | --- | --- |
| No treatment | NA | 0.6503415 | 0.1207890 |
| Before treatment | 0.6503415 | NA | 0.2592995 |
| After treatment | 0.1207890 | 0.2592995 | NA |

**Supplementary Figure 2.** Comparison of sample type (no treatment, vs. pre-treatment vs. post-treatment samples) with regard to alpha diversity (Shannon’s diversity index). NA = not applicable.


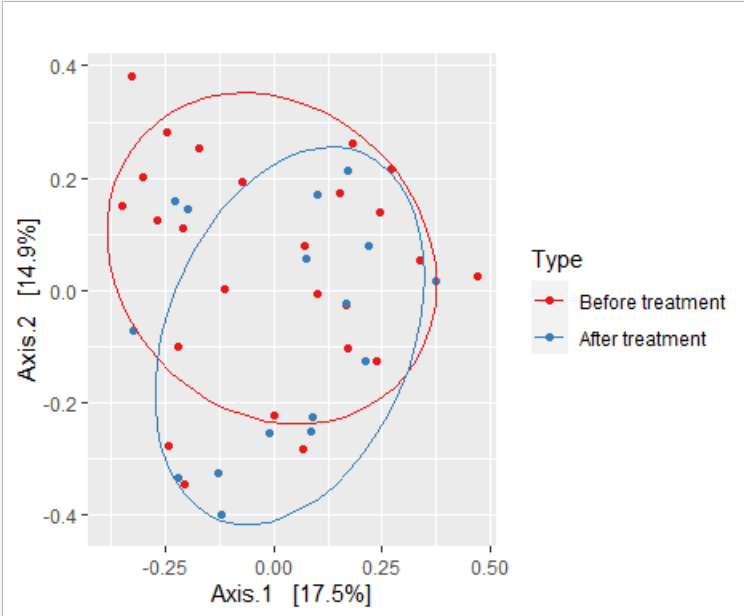


**Supplementary Figure 3.** Comparison of sample type (pre-treatment vs. post-treatment samples) with regard to beta diversity (Bray–Curtis analysis) (*P* = 0.02).


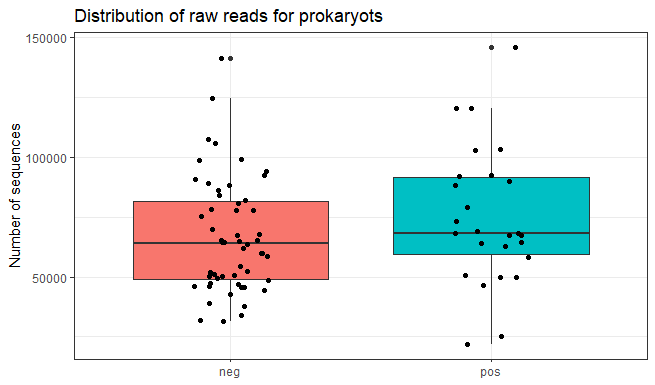


**Supplementary Figure 4.** Comparison of the distribution of raw sequence reads for no-treatment samples only (*P* = 0.15).


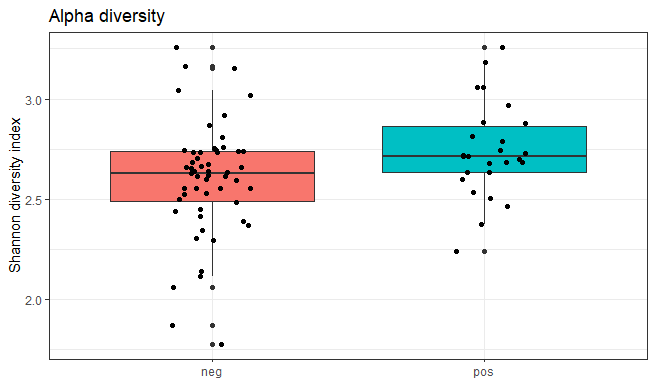


**Supplementary Figure 5.** Comparison of alpha diversity for no-treatment samples only (*P* = 0.03).


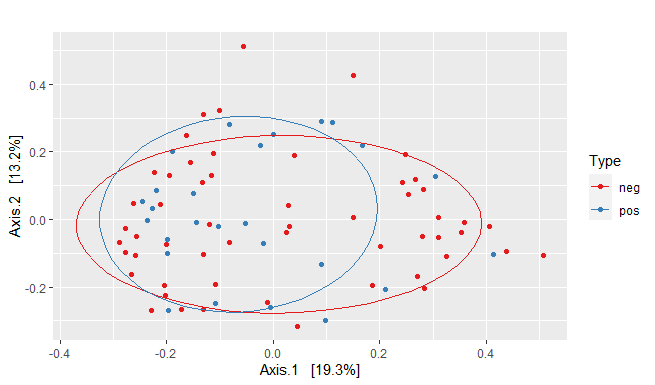


**Supplementary Figure 6.** Comparison of beta diversity in *E. gingivalis*-positive and –negative no-treatment samples only (*P* = 0.90).
